# Beyond tradition: exploring the non-canonical functions of telomeres in meiosis

**DOI:** 10.3389/fcell.2023.1278571

**Published:** 2023-11-13

**Authors:** Alfonso Fernández-Álvarez

**Affiliations:** Institute of Functional Biology and Genomics (IBFG), Consejo Superior de Investigaciones Científicas (CSIC), University of Salamanca, Salamanca, Spain

**Keywords:** telomeres, bouquet, meiosis, gametogenesis, yeast, chromosome dynamics, centromere

## Abstract

The telomere bouquet is a specific chromosomal configuration that forms during meiosis at the zygotene stage, when telomeres cluster together at the nuclear envelope. This clustering allows cytoskeleton-induced movements to be transmitted to the chromosomes, thereby facilitating homologous chromosome search and pairing. However, loss of the bouquet results in more severe meiotic defects than can be attributed solely to recombination problems, suggesting that the bouquet’s full function remains elusive. Despite its transient nature and the challenges in performing *in vivo* analyses, information is emerging that points to a remarkable suite of non-canonical functions carried out by the bouquet. Here, we describe how new approaches in quantitative cell biology can contribute to establishing the molecular basis of the full function and plasticity of the bouquet, and thus generate a comprehensive picture of the telomeric control of meiosis.

## 1 Introduction

The genetic diversity of gametes is facilitated by DNA recombination between homologous chromosomes during meiosis (Petronczki et al., 2003; Hunter, 2015; Zickler and Kleckner, 2015). Strong nuclear movements driven by cytoskeleton motors play a central role in promoting the search and pairing of homologous chromosomes within the nucleoplasm. These movements increase the likelihood of homologous chromosomes meeting and also destabilize interactions between non-homologous chromosomes (Yamamoto et al., 1999; Scherthan et al., 2007; Koszul et al., 2008; Baudrimont et al., 2010; Wynne et al., 2012; Woglar and Jantsch, 2014; Christophorou et al., 2015; Lee et al., 2015; Chacon et al., 2016). For nuclear motion to be transmitted to the chromosomes efficiently, the chromosomes must stay associated with the nuclear envelope (NE). In meiosis, specific associations between telomeres and the NE during the zygotene stage lead to the formation of dynamic clusters of telomeres that are visible through live imaging as groups of telomeres in motion (Chikashige et al., 1994; Klutstein and Cooper, 2014; Mytlis et al., 2023). In some species, these telomere clusters concentrate near a specific region of the NE, often close to the centrosome, resulting in a chromosomal configuration resembling a bouquet of flowers with the telomeres forming the gathered stems. This distinctive meiotic-specific arrangement is thus called the telomere bouquet (Scherthan, 2001). The formation of telomere clusters at the NE, including the telomere bouquet, has been observed in Opisthokonts (fungi and animals) and in plants (Zickler and Kleckner, 2016), suggesting that the origin of telomere bouquet formation is likely contemporaneous with the emergence of the meiotic DNA recombination program in the early evolution of eukaryotes.

For many years, telomere bouquet formation was believed only to facilitate the pairing and subsequent recombination of homologous chromosomes. It was assumed that chromosomes passively followed nuclear movements, with the telomere bouquet acting merely as a spreader of motion. However, loss of the bouquet results in severe defects in meiotic progression that cannot be fully explained by its canonical role (Tomita and Cooper, 2007; Klutstein et al., 2015; Katsumata et al., 2016; Moiseeva et al., 2017). The complete function of the telomere bouquet has remained a mystery, due primarily to the challenge of manipulating and visualizing its transient nature in most eukaryotes (Scherthan, 2001; Fernandez-Alvarez and Cooper, 2017). Advances in quantitative cell biology, coupled with the availability of predictive models and new unsupervised tools based on deep learning for data analysis, now offer opportunities to explore meiotic chromosomal dynamics at high spatial and temporal resolutions. Using these techniques, previously undetectable patterns in telomeric movements have been identified and modelled, providing insights into their biological relevance. From the huge volume of information being generated through these approaches, it is becoming evident that the formation of the telomere clusters in meiosis and the nuclear movements are not random or stochastic. Hence, these recent advances offer exciting opportunities to better understand the molecular basis of the telomeric control of gametogenesis.

## 2 Assembly and disassembly of the telomere bouquet: key to ensuring faithful gametogenesis

The assembly of the telomere bouquet during meiosis coincides with the initiation of the nuclear movements (Yoshida et al., 2013). However, the nuclear movements seem to end before bouquet disassembly (Ruan et al., 2015; Moiseeva et al., 2017). Two components are required for the formation and dissolution of telomere–NE associations: specific telomere bouquet proteins that strengthen the interaction with the NE and promote telomere clustering; and NE proteins that facilitate the interaction with the telomeres, the most common of which is the linker of nucleoskeleton and cytoskeleton (LINC) complex (Hiraoka and Dernburg, 2009; Sosa et al., 2012; Burke, 2018).

The proteins responsible for the meiotic telomere–NE associations are mostly meiotic-specific and have been identified in various organisms: TERB1, TERB2, and MAJIN in mice (da Cruz et al., 2020; Shibuya et al., 2015; Shibuya et al., 2014; Daniel et al., 2014); HIM-8, ZIM-1, ZIM-2, ZIM-3, and MLJ-1 in *Caenorhabditis elegans* (Phillips et al., 2005; Phillips and Dernburg, 2006; Phillips et al., 2009; Kim et al., 2023); Ndj1 and Csm4 in *Saccharomyces cerevisiae* (Conrad et al., 1997; Trelles-Sticken et al., 2000; Conrad et al., 2007; Conrad et al., 2008; Kosaka et al., 2008; Wanat et al., 2008); and Bqt1 and Bqt2 in *Schizosaccharomyces pombe* (Chikashige et al., 2006). However, the sequences of these proteins are not conserved between vertebrates and other metazoans, or even among fungal species. This suggests not only that the proteins responsible for telomere-NE associations have undergone significant turnover during evolution but also that different protein sequences can facilitate the interaction between telomeres and the NE and support bouquet formation. The variability in these protein sequences poses a challenge to the identification of these components in other model organisms that exhibit bouquet formation, such as *Arabidopsis thalian*a.

Telomere bouquet proteins are typically recruited at the telomeres thanks to their direct interaction with shelterin complex (formed by telomere-specific proteins associate with arrays of DNA repeats that protects chromosome ends), which form a protein bridge that connects the telomeres to the LINC complex (de Lange, 2005; Conrad et al., 2007; Hiraoka and Dernburg, 2009; Starr and Fridolfsson, 2010; Rao et al., 2011; Rubin et al., 2020). The LINC complex, which is highly conserved in evolution, plays a crucial role in mediating nuclear movements. The complex consists of a Sad1/UNC-84 (SUN)-domain protein and a Klarsicht, ANC-1, Syne Homology (KASH)-domain protein, both of which interact in the space between the inner and outer nuclear membranes (Hiraoka and Dernburg, 2009). Several studies have demonstrated physical interactions between telomere bouquet proteins (e.g., TERB1/2, Ndj1, and Bqt1) and SUN-domain proteins (e.g., SUN-1/2 in mice, Mps3 in *S. cerevisiae* and Sad1 in *S. pombe*) (Chikashige et al., 2006; Conrad et al., 2007; Conrad et al., 2008; Shibuya et al., 2014). By contrast, the KASH-domain proteins, which are not as highly conserved during evolution as the SUN-domain proteins, interact with cytoskeleton motors in the cytoplasm. Together, these interactions form an intricate network that underpins the orchestration of nuclear movements during meiosis.

Studies in yeast and nematodes have highlighted a strong association between defects in telomere bouquet disassembly and the phosphorylation status of the SUN-domain protein. In budding yeast, the phosphorylation state of Mps3 plays a crucial role in the duration of telomere–NE associations; meiosis-specific phosphorylation introduces negative charges in the luminal region of Mps3, which regulate its localization on the NE for meiotic chromosome motion (Prasada Rao et al., 2021). Phosphorylation of the SUN-domain protein in *C. elegans,* SUN-1*,* is regulated by the widely conserved kinases CDK-1, PLK-2 and CHK-2 (Penkner et al., 2009; Sato et al., 2009; Labella et al., 2011; Woglar et al., 2013; Prasada Rao et al., 2021). In addition, posttranslational modifications of foundational telomere proteins, such as Rap1, may affect their interaction with telomere bouquet proteins; in fission yeast, phosphorylation of Rap1, together with its intrinsic negative charge, control the assembly and disassembly of the bouquet, these features are important for forming interactions with its binding partners Bqt1 and Bqt2 (Amelina et al., 2015).

## 3 Cytoskeleton dynamics in telomere bouquet assembly and disassembly

Actin and dynein are highly conserved motor proteins that have a crucial role in generating forces for nuclear movements during the telomere bouquet stage across various species (Yamamoto et al., 1999; Miki et al., 2002; Koszul et al., 2008; Wynne et al., 2012; Link and Jantsch, 2019). However, the duration, trajectory, and morphology of these nuclear movements vary significantly between species (Rubin et al., 2020; Kim et al., 2022; Sole et al., 2023). For instance, in *S. cerevisiae*, the nuclear membrane undergoes deformations presumably related to rapid telomere-led movements, in which telomeres move in clusters (Hayashi et al., 1998; Scherthan et al., 2007; Conrad et al., 2008). By contrast, in the fission yeast *S. pombe*, the entire nucleus oscillates between the cell poles while the telomeres remain grouped beneath the spindle pole body (SPB), the centrosome equivalent in yeast. This type of movements is commonly referred to as horsetail nuclear movements (Chikashige et al., 1994; Chikashige et al., 2006). Similarly, metazoans demonstrate diverse chromosome morphologies during the telomere bouquet stage: in *C. elegans*, for example, chromatin adopts a crescent shape while being pushed by the nucleolus to one side of the nucleus (Rog and Dernburg, 2013; Rog and Dernburg, 2015; Link and Jantsch, 2019). Conversely, in *Drosophila melanogaster* and mice, characteristic movements involve microtubule-driven chromosomal rotations (Cooley and Theurkauf, 1994; Shibuya et al., 2014). The molecular reasons for the variety of movement types observed in different organisms remain poorly understood. The number of chromosomes could potentially play a role in determining the type of movement. For instance, species with low number of chromosomes, such as fission yeast, may require a more vigorous type of movement. Other factors that could potentially influence movement patterns include the presence of the synaptonemal complex (SC), a structure transiently formed during meiosis to facilitate recombination between homologous chromosomes (Page and Hawley, 2004). Organisms lacking the SC, like *S. pombe* or the ciliate *Tetrahymena thermophila* (Loidl, 2021), may need to employ different dynamics for the movement of their chromosomes compared to organisms with the SC.

The elimination of either actin or dynein, depending on the species, results in the cessation of nuclear movements, which subsequently impedes telomere motions (Miki et al., 2002; Koszul et al., 2008; Wynne et al., 2012). This in turn blocks DNA pairing and recombination, leading to defective chromosome segregation and reduced gamete viability. A meiosis-specific microtubule organizing centre has been identified in certain species, such as *S. pombe* (Saito et al., 2005; Tanaka et al., 2005; Funaya et al., 2012). This microtubule organizing centre, Hrs1, reinforces the dynamic movement of microtubules that is required to pull the SPB back and forth. Loss of Hrs1 results in a slowdown of nuclear movements and, eventually, disassembly of the telomere bouquet.

The formation of the telomere bouquet involves the action of cytoskeleton forces, which cluster the telomeres at specific regions of the NE. In fission yeast, telomere clustering relies on various microtubule motors, kinesins, microtubules and a meiosis-specific microtubule-organizing center named telocentrosome (Yoshida et al., 2013). In particular, the telocentrosome plays a pivotal role in the formation of the telomere bouquet by facilitating the recruitment of the gamma tubulin complex and the movement of telomeres along the NE, from their interphase position, to the SPB (Yoshida et al., 2013). Interestingly, similar structures involving cilia are conserved in zebrafish and mice. These cilia promote the formation of the telomere bouquet by generating microtubule arrays that accumulate at specific regions of the NE (Mytlis et al., 2022).

By contrast, the disassembly of the bouquet appears to be independent of the nuclear movements (Ruan et al., 2015; Moiseeva et al., 2017), suggesting that it occurs after these movements have ended. It is likely that the disassembly of the bouquet is dependent on the completion of other DNA events during meiosis.

## 4 The multifaceted nature of the telomere bouquet

**FIGURE 1:**
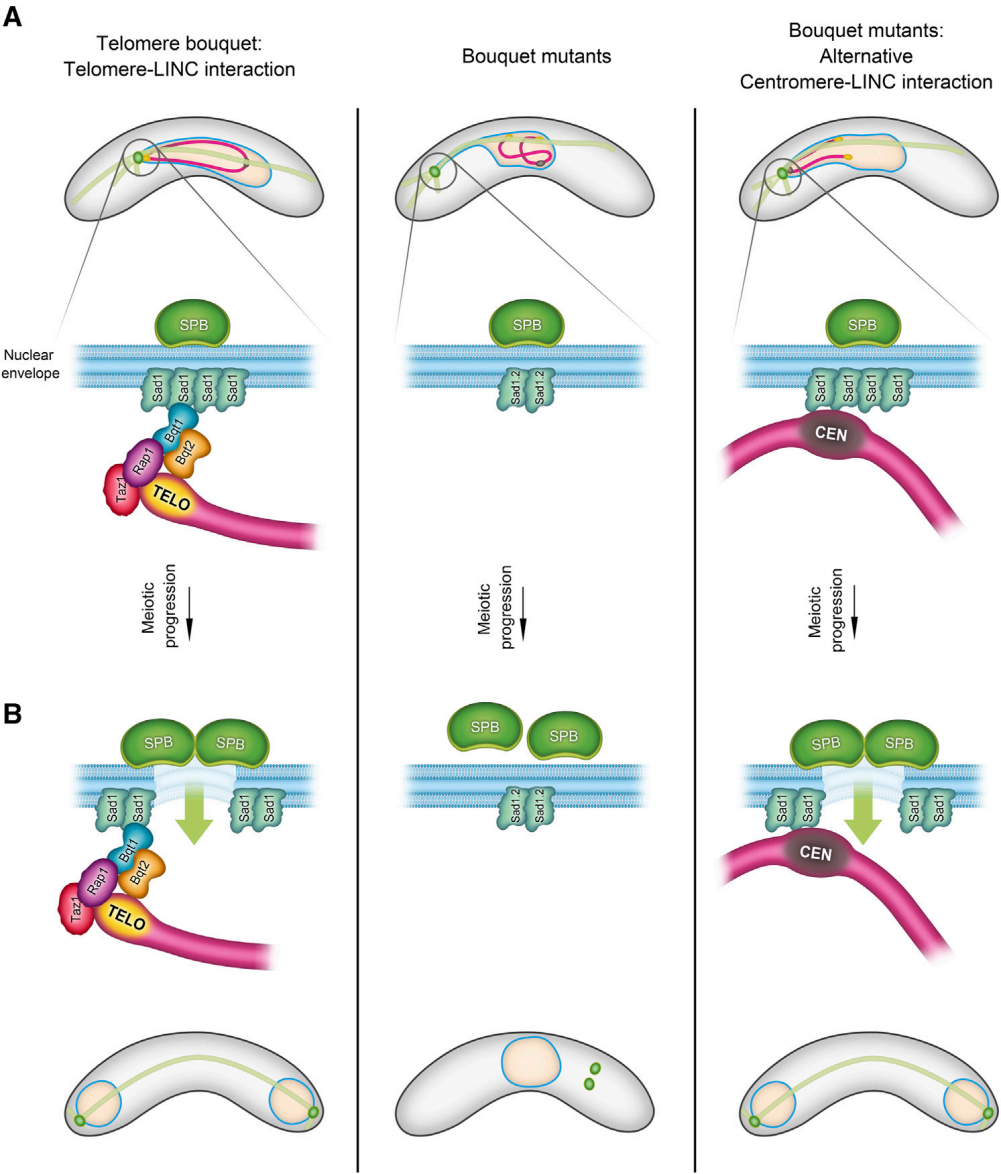
Telomere-Centromere interchange during the bouquet formation stage in *S. pombe.* During the bouquet stage, there is an exchangeability between telomeres and centromeres in their role of facilitating the SPB insertion into the nuclear envelope (NE). The schematic in **(A)** illustrates three scenarios of chromosome-LINC interactions in fission yeast: 1) in normal telomere bouquet formation (left), telomere-LINC interaction enables chromosomes to follow the SPB movements through the interaction of the telomere bouquet protein Bqt1 with the SUN-domain protein, Sad1. 2) In bouquet mutants (right), where *bqt1* and/or *bqt2* are deleted, interaction between telomeres and Sad1 is disrupted. However, centromeres are capable of contacting Sad1, forming an alternative “centromere” bouquet conformation. 3) The middle panel shows a combined scenario, *bouquet∆* in conjunction with the Sad1.2 allele, resulting in the inhibition of both telomere-LINC and centromere-LINC interactions. In **(B)**, the left panel depicts the triggering of partial NE disassembly by telomere-LINC interaction, facilitating the SPB insertion into the NE and spindle formation. This signalling can also be controlled by the centromeres (right). Lack of interaction between telomeres/centromeres and the LINC complex disrupts the SPB insertion process, thereby compromising spindle formation (middle panel) (more information in (Fennell et al., 2015; Fernández-Álvarez et al., 2016).

Several studies–particularly in fission yeast, where live imaging allows for a more detailed analysis–have revealed unexpected functions of the telomere bouquet. For instance, the Cooper and Yamamoto labs have shown that the absence of bouquet formation compromises the formation of spindle microtubules, which are crucial for chromosome segregation (Tomita and Cooper, 2007; Katsumata et al., 2016). Elimination of telomere bouquet proteins such as Bqt1 or Bqt2 leads to defects in spindle formation and thus to aberrant chromosome segregation. These defects are associated with problems in the localized NE disassembly, a process that necessitates the proximity of telomeres to create a hole in the NE for the insertion of the duplicated SPBs. This stage of NE disassembly beneath the SPB is analogous to the NE breakdown stage observed in mammals (Fernández-Álvarez et al., 2016) (Figure 1). The proximity of telomeres to the NE likely triggers a modification in the SUN-domain protein, Sad1, in *S. pombe*, leading to the reorganization of Sad1 to form a ring, which in turn promotes local NE disassembly and SPB insertion (Fernández-Álvarez et al., 2016; Bestul et al., 2017).

The formation of the telomere bouquet controls another crucial function in the meiotic program: centromere reassembly. During meiosis, centromeres must be disassembled in preparation for specialized chromosome segregation in the first round of nuclear division. During bouquet formation, a microenvironment is created around the SPB that is characterized by the proximity of centromeres and telomeres, resulting in the transfer of heterochromatin factors from the telomeres to the centromeres (Klutstein et al., 2015; Hou et al., 2021).

Moreover, the Hiraoka and Tomita labs discovered that defects in DNA replication and repair prolong the duration of nuclear movements and the telomere bouquet stage in fission yeast (Ruan et al., 2015; Moiseeva et al., 2017). This finding indicates that bouquet assembly and disassembly are coordinated with crucial chromosomal events. Furthermore, we have found that DNA repair may affect not only the duration of the bouquet but also the behaviour of telomeric movements during meiosis. Specifically, persistent DNA damage alters the trajectory of telomeres during the horsetail movement, likely facilitating DNA repair between homologous chromosomes to ensure accurate meiotic progression (Leon-Perinan and Fernandez-Alvarez, 2020; Leon-Perinan and Fernandez-Alvarez, 2021).

## 5 Alternative conformations of the telomere bouquet and their evolutionary significance

In addition to being studied extensively in Opisthokonts, telomere bouquet formation has been identified in species of the Chloroplastida and Alveolata groups, indicating its likely evolution from the origin of eukaryotes along with the meiotic program (Scherthan, 2001; Zickler and Kleckner, 2016; Hurel et al., 2018). Although bouquet formation is conserved in evolution, it displays some conformational plasticity, which has led to variations in the number and distribution of telomere clusters along the NE that in turn result in differences in chromosome polarization and the trajectories of telomere movements between species (Rubin et al., 2020; Sole et al., 2023). Notably, two common variations involve the diversity of the meiosis-specific telomere protein sequences that support telomere-NE associations (Rubin et al., 2020; Kim et al., 2022) and the unexpected interchangeability between telomeres and centromeres. In certain scenarios, a so-called centromere bouquet can replace the telomere bouquet (Stewart and Dawson, 2008; Loidl et al., 2012; Fennell et al., 2015) (Figure 1).

As described above, loss of the telomere bouquet in fission yeast causes severe defects in local NE disassembly and, consequently, in the SPB insertion into the NE and spindle formation (Tomita and Cooper, 2007; Pineda-Santaella and Fernández-Álvarez, 2019; Pineda-Santaella et al., 2021). However, approximately 50% of bouquet-mutant cells can form normal spindles by using centromeres in prophase to create a bouquet-like structure (Fennell et al., 2015) (Figure 1). Given that centromeres and telomeres represent distinct chromosomal regions, the common features that support this capacity for substitution have yet to be uncovered. The molecular bases that underpin this interchangeability are intriguing, given that telomeres and centromeres do not commonly share functions.

In the protist *T. thermophila*, the nucleus undergoes substantial stretching in meiotic prophase, with chromosomes adopting a bouquet-like arrangement in which telomeres and centromeres attach to opposite poles of the nucleus. Centromere clustering was found to be more important than telomere clustering for homologous pairing, suggesting that centromere clustering may have been the primordial mechanism for chromosome pairing (Tian et al., 2020). In *D. melanogaster*, it is the centromeres rather than the telomeres that support the formation of the bouquet (Rubin et al., 2020). It remains an enigma as to why telomeres perform this function in certain organisms while centromeres assume this role in others. This intriguing and unconventional nature of these occurrences raises the question of whether it is of significance whether it is telomeres or centromeres carrying out these functions.

Another question that we are currently exploring is how to cluster telomeres at the NE without the highly conserved LINC complex. Whereas the sequence of telomere bouquet proteins may be highly divergent between species, the presence of the LINC complex–which has a crucial role in transmitting movement to the chromosomes–has remained highly conserved since the origin of eukaryotes. We have found that in some Basidiomycota fungi, such as the pathogen *Ustilago maydis*, all the machinery of the meiotic recombination program and the telomeric proteins (e.g., Taz1 and Rap1) are conserved (Kojic et al., 2013), but the LINC complex is missing. This raises questions about which elements are essential for bouquet formation and which have undergone more turnover throughout evolution. Finding the answers to these questions will help us to determine whether meiotic chromosome movements and the formation of the telomere bouquet have driven the evolution of the meiotic program.

## 6 New imaging techniques provide insights into telomere motion

Both the canonical and non-canonical functions of the telomere bouquet are closely related to the chromosomal conformations during this stage. The canonical function involves transmitting forces generated in the cytoplasm through the movement of the telomere clusters along the NE (Scherthan, 2001; Zickler and Kleckner, 2016; Mytlis et al., 2023). By contrast, the non-canonical functions of the bouquet as a regulator of meiotic spindle formation or centromere assembly require the telomeres to be in close proximity to the NE. This is so that the localized NE disassembly can be triggered, which is necessary for proper spindle formation (Tomita and Cooper, 2007; Fernández-Álvarez et al., 2016), or to create the microenvironment that supports centromere reassembly during meiotic prophase (Klutstein et al., 2015; Hou et al., 2021). We have observed that telomere trajectories along the NE during bouquet stage in fission yeast are not stochastic but instead follow movement patterns that are imperceptible by direct human observation but are computationally identifiable and mathematically predictable (Leon-Perinan and Fernandez-Alvarez, 2021). Hence, telomere movements along the NE change trajectory and velocity in response to specific chromosomal events, such as DNA repair. Tracking this behaviour in detail is key to understanding the functions of telomere clustering and to uncovering new connections to meiosis.

Many studies have investigated recognizable chromosome movement patterns using tracking schemes to monitor chromosome behaviour in organisms such as *S. cerevisiae* (Scherthan et al., 2007; Conrad et al., 2008; Gonzalez-Arranz et al., 2020), *C. elegans* (Baudrimont et al., 2010; Wynne et al., 2012; Labrador et al., 2013; Woglar and Jantsch, 2014; Rog and Dernburg, 2015) and *S. pombe* (Ding et al., 2004; Chacon et al., 2016; Moiseeva et al., 2017). Time-lapse fluorescence microscopy is commonly used to follow the movements of particles, including proteins like dynein, as well as chromosomal loci (Mine-Hattab and Rothstein, 2012; Ananthanarayanan et al., 2013). Methods such as mean square displacement, velocity measurements, and automatic and cross-correlation analyses have been used to evaluate long-range spatiotemporal patterns, generating a high volume of information about chromosome dynamics at specific loci (Mine-Hattab and Chiolo, 2020). For example, these approaches have been used in budding yeast to identify and characterize rotational meiotic movements that result from both nuclear rotation and individual chromosome movements (Lee et al., 2015). Studies in human cells have shown that chromosome end motion is both highly heterogeneous and inversely related to telomere length (Wang et al., 2008) and that telomeres display intermittent accumulations in specific local niches that depend on their exposure to different types of stress (Benelli and Weiss, 2022). One of the most relevant findings in recent years is the observation that upon exposure to DNA-damaging agents, telomeres are more likely to move away from their sites on the NE. These discoveries demonstrate that a combination of factors, including the release of chromatin-NE tethering, internal chromatin connections, and microtubule dynamics, work together to mobilize the genome in response to DNA damage (Therizols et al., 2006; Mine-Hattab and Rothstein, 2012; Lawrimore et al., 2017; Mine-Hattab and Chiolo, 2020).

These findings, together with the optimization of model organisms for visualizing chromosome dynamics, such as zebrafish (Blokhina et al., 2019; Imai et al., 2021; Mytlis et al., 2022; Mytlis et al., 2023) and *Arabidopsis thaliana* (Hurel et al., 2018), are paving the way for exciting new research opportunities in this field.

However, these types of techniques have limitations arising from their time-ensemble nature (Mine-Hattab and Chiolo, 2020). For example, different modes of motion can produce the same mean square displacement curves or velocity distributions, since trajectories that are effectively different can nevertheless produce identical distribution summaries. This means that specific patterns of chromosome movements, particularly those not yet linked to a known biological process, cannot be easily identified. Hence, complementary strategies to explore chromosome dynamics are being developed. For example, novel imaging techniques with a low signal-to-noise ratio offer exciting prospects for further investigations into homologous pairing (Nozaki et al., 2021). Correlative conventional and PALM (photoactivated localization microscopy) imaging enhances our capacity to analyse the mobility and time-averaged nanoscopic structural characteristics of locus-specific chromatin with single-molecule sensitivity (Mehra et al., 2022). Using point-spread-function engineering and deep-learning-based image analysis, we can now conduct live imaging of telomere diffusion (Naor et al., 2022).

## 7 Harnessing data mining and causality analysis for predictive modelling

A major limitation in developing a predictive model is the need for large sample sizes. Data mining and time-window approaches offer solutions to some of these limitations. For example, researchers are now automatically creating synthetic variations of chromosome movements during the telomere bouquet stage based on wild-type and mutant datasets. The creation of *in silico* versions of budding yeast strains and their analysis using experimental data and simulations have revealed important information about the active motion of telomeres and the biological implications of the bouquet (Penfold et al., 2012; Marshall and Fung, 2016; Marshall and Fung, 2019; Navarro et al., 2022). For example, this approach has revealed that active telomere forces can increase the selectivity of chromosome pairing (Marshall and Fung, 2019). Complementary approaches are using segment-discovery libraries, like segclust2d and segmenTier, and matrix profile calculations to extract information about chromosome movement from time-lapse experiments. At the same time, causality analysis algorithms, such as Peter-Clark algorithm, variable-lag transfer entropy and variable-lag Granger causality, can be used to identify whether changes in one variable (e.g., chromosome morphology) affect another variable (e.g., chromosome movement) (Leon-Perinan and Fernandez-Alvarez, 2021). As these algorithms help to establish causal relationships, they provide valuable information for understanding the mechanisms and regulation of chromosome dynamics during meiotic prophase in various organisms.

## 8 Summary

**FIGURE 2:**
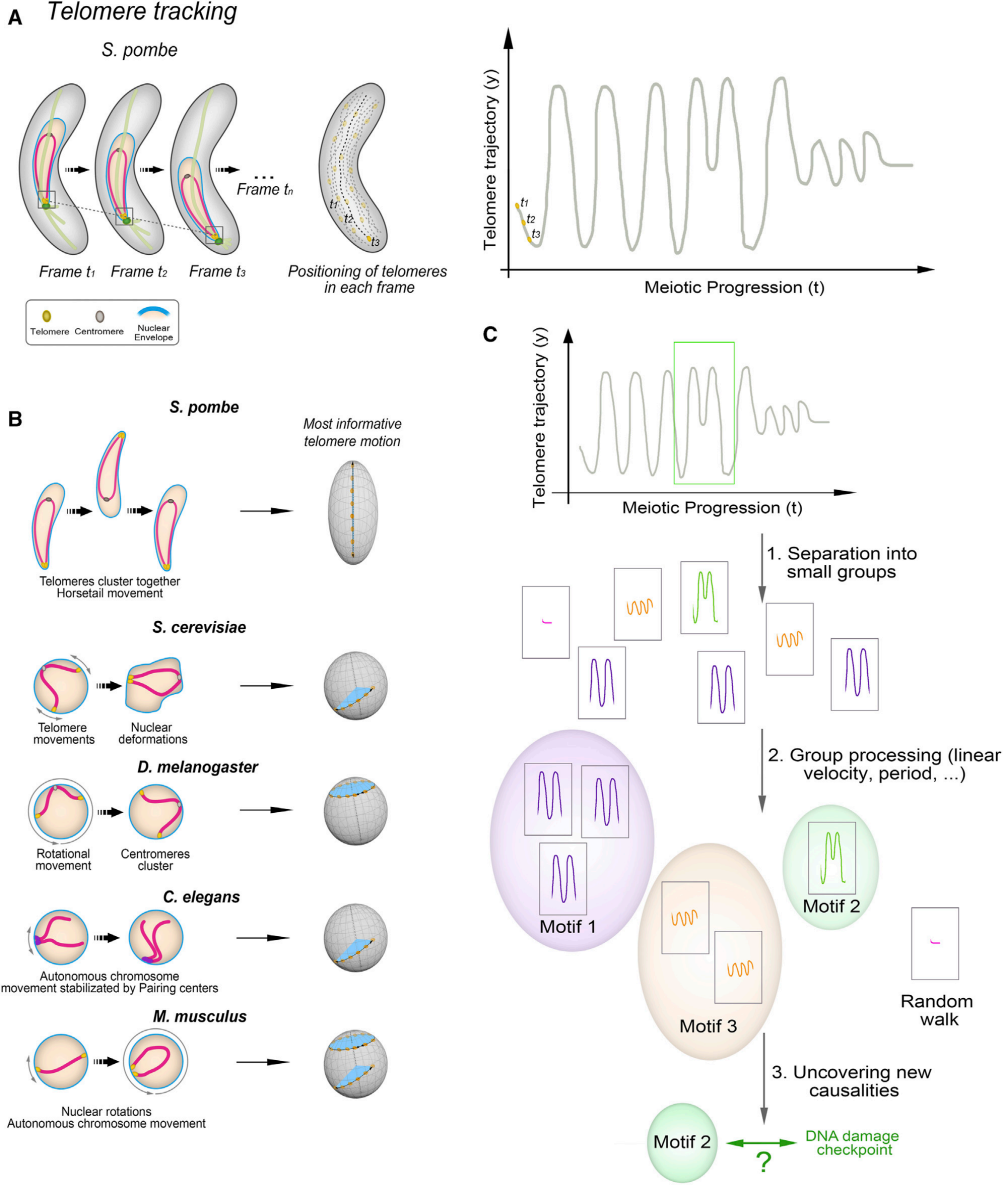
Unveiling Telomere Movement Patterns in Fission Yeast and Prospects for Cross-Species Applications **(A)** Illustration depicting the process employed to analyze telomere positioning in fission yeast. Quantification and tracking of telomere positioning, representation of telomere motion in the *y*-axis, the most informative in case of *S. pombe*. **(B)** Zygotene-stage telomere movements exhibit distinct characteristics across species. Linear movements covering short distances along the nuclear envelope (NE) are observed in *S. cerevisiae* and *C. elegans*, while *D. melanogaster* displays rotational movements. *M. musculus* displays both types. **(C)** Outline of the process used to identify telomere movement patterns. Key steps involve determining the primary axis of movement—such as the *y*-axis for fission yeast’s oscillatory motion—tracking trajectories via *in vivo* telomere labelling, segmenting time intervals, and clustering based on various variables like linear/angular velocity and period. Comparisons between wild-type and mutant strains unveil the presence or absence of ‘motifs’ and their distribution throughout prophase.

The telomere bouquet is conserved in eukaryotes and has both canonical and non-canonical functions. Its canonical functions involve transmitting the forces generated in the cytoplasm to promote the chromosome movements needed to facilitate homologous pairing, while its non-canonical functions include regulating meiotic spindle formation, meiotic centromere assembly and DNA events such as replication and repair. New techniques–including time-lapse fluorescence microscopy, tracking schemes, and data mining–are now enabling researchers to circumvent the limitations of previous experimental approaches. These techniques have been used to identify patterns of chromosome movement, such as rotational meiotic movements, and modifications to the trajectory of chromosomes in response to DNA events (Figure 2). Combining these techniques with causality analysis algorithms and other advances in quantitative cell biology, such as low-signal-to-noise imaging and deep-learning-based analysis, offers opportunities to explore chromosomal motion at even higher spatial and temporal resolutions. These techniques offer new insights into homologous pairing and nanoscopic structural features of chromatin.

## Data Availability

The raw data supporting the conclusion of this article will be made available by the authors, without undue reservation.
